# Use of Magill Forceps to Remove Foreign Bodies in Children

**DOI:** 10.1055/s-0037-1604102

**Published:** 2017-06-19

**Authors:** Murat Oncel, Guven Sadi Sunam, Cagdas Elsurer, Huseyin Yildiran

**Affiliations:** 1 Department of Thoracic Surgery, Medical Faculty, Selcuk University, Konya, Turkey; 2 Department of Otorhinolaryngology, Medical Faculty, Selcuk University, Konya, Turkey

**Keywords:** Magill forceps, foreign bodies, methods

## Abstract

**Introduction** Esophageal foreign body (FB) in all age groups can cause serious morbidity or mortality. The study aims to report our experience retrieving FBs from the upper esophagus in children using Magill forceps.

**Materials and Methods** In this study, 88 patients (45 males [51.1%] and 43 females [48.9%]) were presented with suspected FB ingestion. FB ingestion was determined via endoscopic analysis, or lateral and posterior–anterior radiographies, including oropharynx, neck, chest, and abdomen. Cases were classified into seven groups, according to history, diagnostic method, and postintervention findings, as follows: (1) coins, (2) toys, (3) metals, (4) bones, (5) battery, (6) glass, and (7) food. A laryngoscope was used to elevate the larynx and expose the esophageal entrance. Magill forceps were advanced into the esophagus and opened to observe and extract the FB.

**Results** All 88 patients who underwent endoscopic examination due to suspected FB ingestion were confirmed to have ingested a FB. Median age was 12 years; 15 patients were aged < 5 years; 63 (71.5%) were diagnosed based on routine radiographic findings, and others were diagnosed based on physical findings and history. The most common type of FB was coins ( *n* = 51 [57.9%]). Mean surgical duration was 20 minutes.

**Conclusion** FBs located at cervical esophageal level are usually the most difficult to remove. Magill forceps should be used before other methods.

Accidental foreign body (FB) ingestion is a common clinical problem. 1 FB ingestion is highly prevalent among the pediatric age group. In adults, it occurs most frequently in alcoholics, prisoners, and those with mental retardation. 2 3 Radiological localization of ingested FB using advanced techniques is mandatory. 4 Esophagoscopy is the main method for the removal of FBs. Rigid esophagoscopy has been mainly associated with a 5 and 10% risk of perforation during FB removal. The ideal methods are all of the procedures which have lower perforation rate to quickly remove the FBs. Foley catheter extraction and the minimally invasive Magill forceps devices were described for this goal to remove FBs which located in the upper esophagus. The present study aimed to report our experience retrieving ingested FBs from the upper esophagus in children using Magill forceps under general anesthesia.

## Materials and Methods

**Fig. 1:**
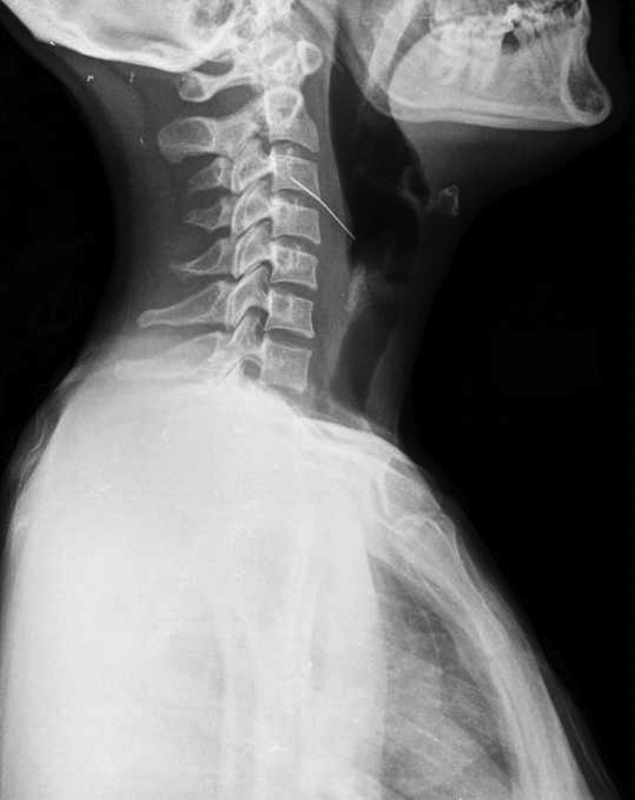
Lateral view shows a turban pin in the first part of the esophagus.

In total, 88 patients (45 males [51.1%] and 43 females [48.9%]) presented with suspected FB ingestion to the School of Medicine Hospital and Konya State Hospital, Selcuk University between January 1996 and July 2015. FB ingestion was determined via endoscopic analysis, or lateral and posterior–anterior radiographies, including the oropharynx, neck, chest, and abdomen. Cases of FB ingestion were classified into seven groups, according to history, diagnostic method, and postintervention findings, as follows: (1) coins, (2) toys, (3) metals, (4) bones, (5) battery, (6) glass, and (7) food ( Figs. 1 2 3 4 5 6 7 ). None of the patients had a history of esophageal disease. Clinical symptoms, such as dysphagia, pain when swallowing, and excessive salivation, were recorded. Excessive salivation that occurred suddenly after ingestion of an FB was the strongest diagnostic criterion. All cases of FB ingestion were considered emergencies, and interventions were performed immediately in the surgical suite. The patients were sedated and FBs were extracted was using Magill forceps, without intubation. A laryngoscope was inserted into the pharynx to elevate the larynx and to expose the esophageal entrance. Magill forceps were advanced into the esophagus and opened to observe and extract the FB. General anesthesia was administered using sevoflurane in a mixture of oxygen/nitrous oxide (50:50) via mask ventilation. For procedures that exceeded 20 minutes, dexamethasone was administered to prevent soft tissue edema. Postprocedure each patient underwent follow-up X-ray and the patients' parents were advised to prohibit their child from eating and drinking for 8 to 10 hours. The children were generally discharged the same day as the procedure.

**Fig. 2:**
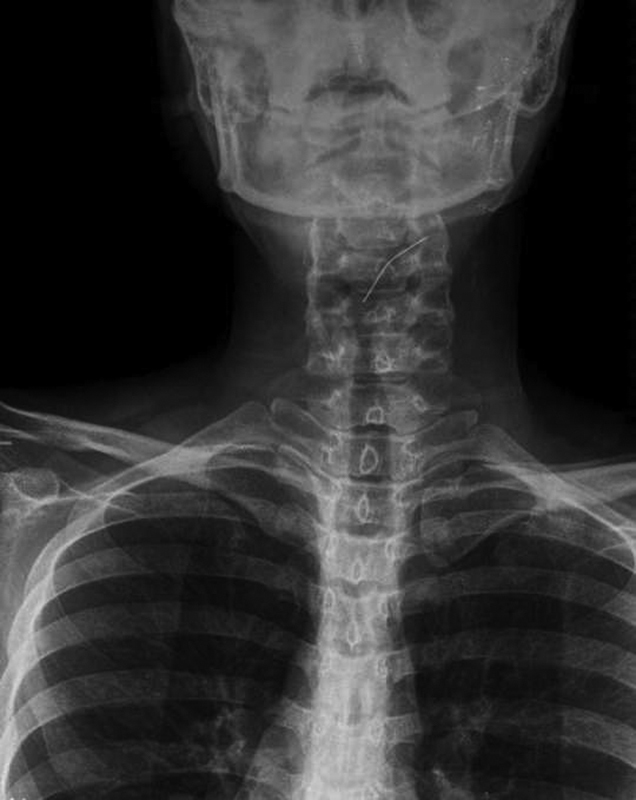
Anterior view shows a turban pin in the first part of the esophagus.

**Fig. 3:**
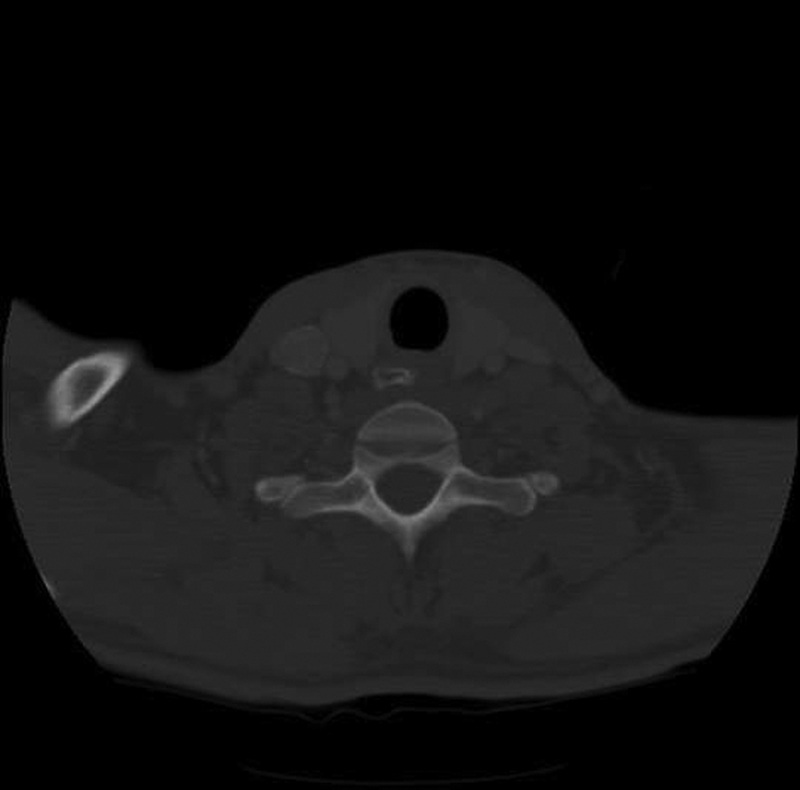
Cervical computed tomography shows a chicken bone in the first narrowing of the esophagus.

**Fig. 4:**
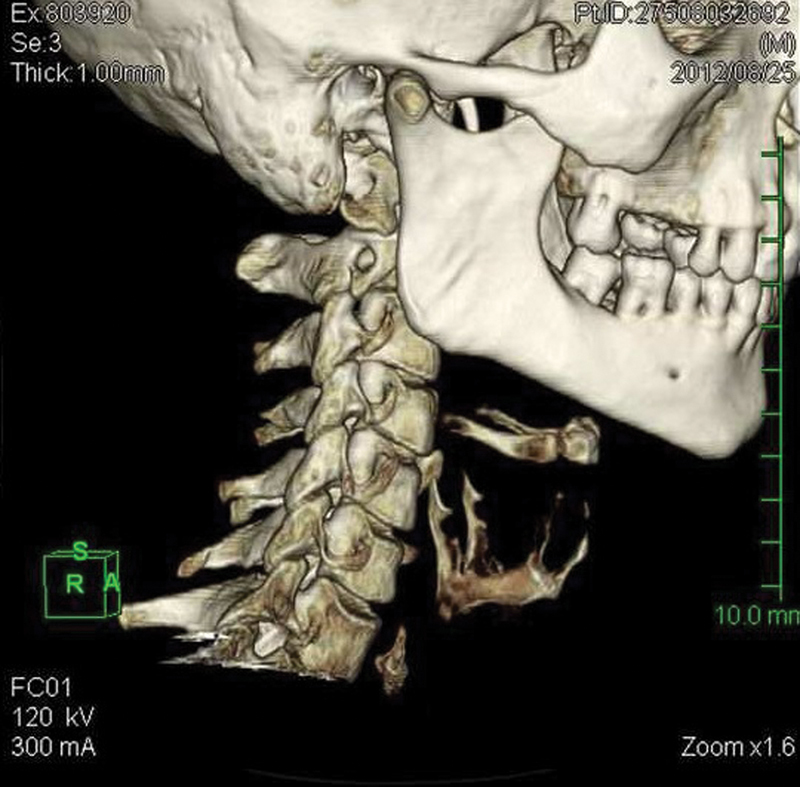
Three-dimensional view shows a bone in the first narrowing of the esophagus (same patient as in Fig. 3 ).

**Fig. 5:**
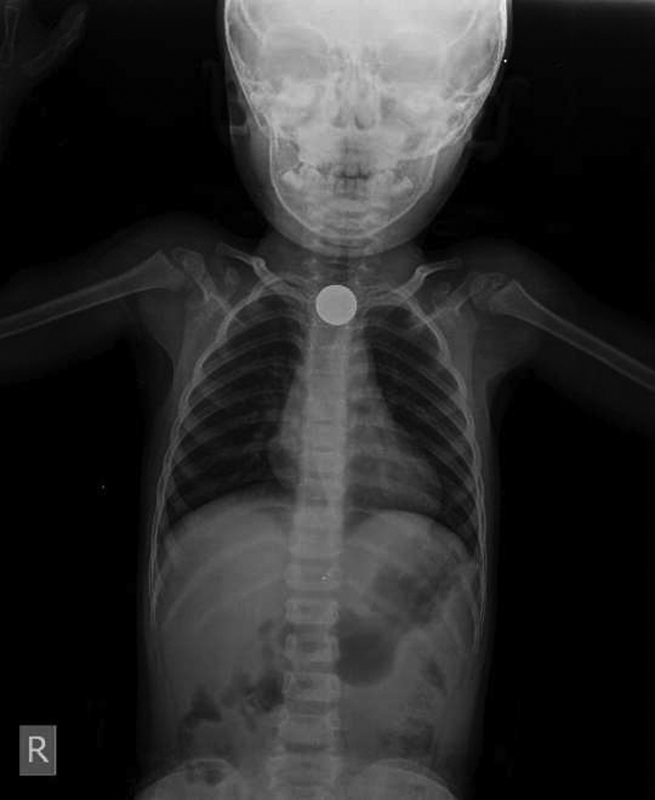
X-ray shows a coin in the first part of the esophagus.

**Fig. 6:**
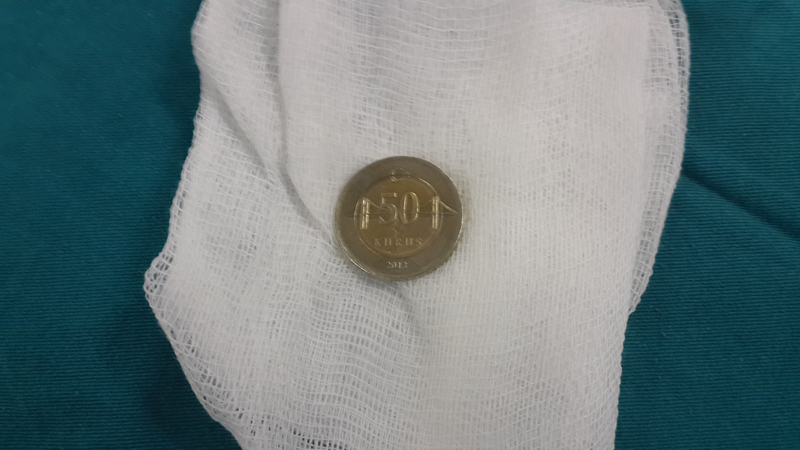
Coin removed from the first narrowing of the esophagus.

**Fig. 7:**
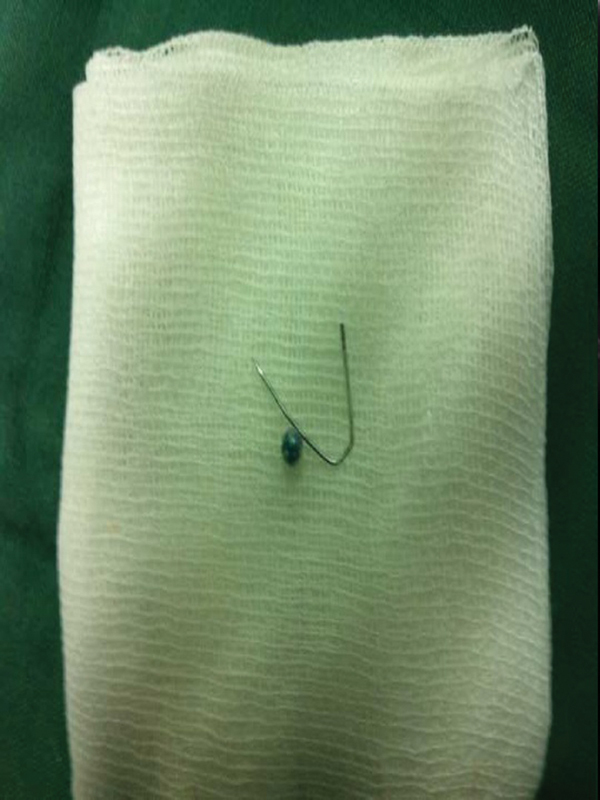
A turban pin removed from the first narrowing of the esophagus via Magill forceps.

## Results

Of the 88 patients who underwent endoscopic examination due to suspected FB ingestion, all were confirmed to have ingested an FB. Median age of the patients was 12 years; in all, 15 patients were aged < 5 years ( Table 1 ). Among the patients, 63 (71.5%) were diagnosed based on routine radiographic findings; the other 25 were diagnosed based on physical findings and FB history. None of the patients had esophageal disease, such as esophageal stenosis secondary to corrosive injury or congenital esophageal atresia. In all, two patients had cerebral palsy and mental retardation. The most common type of FB ingested was coins ( *n* = 51 [57.9%]) ( Table 2 ). Diameter of coins ranged from about from 20 to 26 mm. The length of pins which had a sharp point at one end and a round plastic head at the other end was ∼30 mm. The diameters of batteries ranged from 10 to 20 mm. The pieces of food and toy had different sizes and lengths and especially the one end of bones had sharp point. The most common symptom of FB ingestion was hypersalivation ( *n* = 61 [69.3%]) due to inability to swallow. The other symptoms of patients included discomfort in the throat, dysphagia, and difficulty in speaking. Almost all FBs were removed via a single procedure. Mean surgical duration was 20 minutes (range: 15–45 minutes). In six (6.8%) of the patients who ingested a coin, the coin passed into the digestive tract while attempting to remove it from the upper region of the esophagus; none of these patients had any complications and all the coins were recovered from stool. Especially, if the FB was a coin, we stopped the oral feeding for 24 hours. Also, we did not see any complications in that patients.

**Table 1:** Age distribution of foreign body

Age (y)	Coin	Toys	Metal	Battery	Bone	Glass	Food	Total
**0–3**	7	–	1	2	–	–	–	10
**4–7**	22	5	1	–	–	–	1	29
**8–11**	16	3	1	1	3	–	–	24
**12–22**	6	–	11	–	5	–	–	22
More than **22**	–	–	–	–	1	1	1	3
**Total**	51	8	14	3	9	1	2	88

**Table 2:** Nature and frequency of foreign bodies ingested

Type of foreign bodies	Number of patients ( *n* , %)
**Coin**	51 (57.9)
**Food bolus**	2 (2.3)
**Fish bones**	2 (2.3)
**Chicken bones**	7 (8)
**Turban pins**	13 (14.8)
**Toys**	9 (10.2)
**Glass**	1 (1.1)
**Battery**	3 (3.4)
**Total**	88 (100)

## Discussion

Esophageal FB is an emergency clinical condition that occurs in all age groups (most commonly in children) that can cause serious morbidity or mortality. FBs retained in the esophagus generally fall into two categories: FB and food bolus. Children most often ingest coins, toys, and metal objects, whereas adults commonly present with meat bolus and bones. 4 More importantly, physical and mental conditions predispose patients to esophageal impaction. FBs located near the cervical esophageal level are usually the most difficult to remove, and based on our clinical experience, Magill forceps should be used before resorting to other methods. Rapid diagnosis and treatment are important to minimize morbidity and complications associated with FB ingestion. 5 Impacted FBs in the esophagus can easily cause mucosal ulceration and even infection, and can also result in various fatal complications, including retroesophageal abscess, mediastinitis, and empyema. 6 The aim of initial patient evaluation is to identify the type of FB and its location, and to determine if there are any underlying esophageal conditions. Radiographic evaluation is helpful for confirming the location of an FB and associated complications. Plain radiography of the neck and chest commonly show the location of radiopaque objects, such as coins, turban pins, and metallic objects. Both anterior–posterior and lateral views are necessary for the diagnosis. 7 Nonradiopaque FBs must be removed with great caution; however, there is a lack of consensus regarding the best method for doing so. The choice of treatment is determined based on many factors, including patient age and clinical condition, the level of emergency, and the size of the ingested FB and its anatomic location. Esophageal FBs account for 7 to 14% of all esophageal perforations, and fish and chicken bones are the most common FBs in adults. 8 Sharp objects and batteries should be removed as soon as possible to prevent esophageal erosion or necrosis of the mucosa; 2 however, Eisen et al suggest that small batteries (< 20 mm in diameter) that have passed beyond the esophagus need not be retrieved unless patients have gastrointestinal (GI) tract symptoms and signs, such as abdominal pain, abdominal distension, or vomiting. 3 Patients may be symptomatic immediately following ingestion of an FB or as late as 2 weeks after esophageal perforation. 9 Symptoms of FB ingestion include dysphagia, odynophagia, and chest pain. If an FB forms a complete obstruction, patients exhibit sialorrhea and regurgitation. Up to 5% of patients can present with airway obstruction when a bolus is impacted near the upper esophageal sphincter because there is compression of the trachea that causes symptoms of stridor and coughing. 10 The most common symptoms in the present study's patients were odynophagia and chest pains. Chest X-rays may show perforation-related complications, such as air and fluid collection, or abscess in the pleural space, pericardium, or mediastinum; however, a chest X-ray can show normal findings in some cases and alone is not adequate for detecting an FB retained in the esophagus. The most important consideration, especially in children, is airway control. 2 Endoscopy is currently the most widely used method of FB removal; 11 its advantages are direct observation and evaluation of the degree of esophageal injury caused by an FB. Rigid esophagoscopy had been the primary tool for the removal of FBs until 1957, when Hirschowitz constructed the first flexible fiber-optic endoscope employed by gastroenterologists for investigating patients with complaints involving the upper digestive tract. 12 Today, rigid and flexible endoscopy are performed under general anesthesia and conscious sedation, respectively. Magill forceps are considered safe and effective in experienced hands. In the present study, coins and other FBs were extracted from the upper esophagus using Magill forceps, and we recommend this technique for easy removal of FBs without the need for deep general anesthesia and/or intubation. This method was used to extract coins that passed down to the first constriction of the esophagus, as well as to extract food and other FBs. In cases in which the FB passed into the stomach, we recommended the patient wait for its passage in stool; 11 however, abdominal pain may be an indication for surgical removal. Children's natural curiosity and propensity to ingest objects are well known and supported by X-rays and a history of FB ingestion. Also, it is very important that parents should be aware of complications of esophageal FBs. 13

In conclusion, the presence of an FB in the esophagus is a challenging problem, as perforations may result in death. Rapid diagnosis and treatment of FBs in the esophagus will decrease the morbidity rate and duration of hospitalization. 14 There are many options available for the management of esophageal FBs. Our clinical experience indicates that FBs located in the upper esophagus should be removed using Magill forceps. This approach is the preferable method for extraction of upper-GI FBs because of its high success rate without complications. Early diagnosis and detection of FBs are essential for easy removal. Coins and other radiopaque items can be diagnosed via X-ray and safely removed using direct vision via Magill forceps while patients are sedated.

## References

[1] Arana A Hauser B Hachimi-Idrissi S Vandenplas Y Management of ingested foreign bodies in childhood and review of the literature Eur J Pediatr 2001 160 08 468 472 11548183 10.1007/s004310100788

[2] Webb W A Management of foreign bodies of the upper gastrointestinal tract: update Gastrointest Endosc 1995 41 01 39 51 7698623 10.1016/s0016-5107(95)70274-1

[3] Eisen G M Baron T H Dominitz J A Guideline for the management of ingested foreign bodies Gastrointest Endosc 2002 55 07 802 806 12024131 10.1016/s0016-5107(02)70407-0

[4] Kim J K Kim S S Kim J I Management of foreign bodies in the gastrointestinal tract: an analysis of 104 cases in children Endoscopy 1999 31 04 302 304 10376456 10.1055/s-1999-13

[5] Schunk J E Corneli H Bolte R Pediatric coin ingestions. A prospective study of coin location and symptoms Am J Dis Child 1989 143 05 546 548 2718987

[6] Singh B Kantu M Har-El G Lucente F E Complications associated with 327 foreign bodies of the pharynx, larynx, and esophagus Ann Otol Rhinol Laryngol 1997 106 04 301 304 9109720 10.1177/000348949710600407

[7] Pinto A Muzj C Gagliardi N Role of imaging in the assessment of impacted foreign bodies in the hypopharynx and cervical esophagus Semin Ultrasound CT MR 2012 33 05 463 470 22964412 10.1053/j.sult.2012.06.009

[8] Kanowitz A Markovcick V Esophageal and diaphragmatic trauma St Louis Mosby 1998 546 548

[9] Silva R G Ahluwalia J P Asymptomatic esophageal perforation after foreign body ingestion Gastrointest Endosc 2005 61 04 615 619 15812424 10.1016/s0016-5107(05)00081-7

[10] Duncan M Wong R K Esophageal emergencies: things that will wake you from a sound sleep Gastroenterol Clin North Am 2003 32 04 1035 1052 14696296 10.1016/s0889-8553(03)00087-6

[11] Al-Qudah A Daradkeh S Abu-Khalaf M Esophageal foreign bodies Eur J Cardiothorac Surg 1998 13 05 494 498 9663527 10.1016/s1010-7940(98)00068-2

[12] Hirschowitz B I A personal history of the fiberscope Gastroenterology 1979 76 04 864 869 369936

[13] Gregori D Scarinzi C Morra B Ingested foreign bodies causing complications and requiring hospitalization in European children: results from the ESFBI study Pediatr Int 2010 52 01 26 32 19419514 10.1111/j.1442-200X.2009.02862.x

[14] Messner A H Pitfalls in the diagnosis of aerodigestive tract foreign bodies Clin Pediatr (Phila) 1998 37 06 359 365 9637900 10.1177/000992289803700605

